# Protective effect of lithium pyruvate against oxidative damage to peripheral blood mononuclear cells

**DOI:** 10.1192/j.eurpsy.2024.507

**Published:** 2024-08-27

**Authors:** L. Smirnova, E. Epimakhova, E. Plotnikov, I. Losenkov

**Affiliations:** ^1^Laboratory of Molecular Genetics and Biochemistry, Mental Health Research Institute, Tomsk National Research Medical Center of the Russian Academy of Sciences; ^2^Division of Biology and Genetics, Siberian State Medical University; ^3^Research School of Chemistry & Applied Biomedical Sciences, Tomsk Polytechnic University, Tomsk, Russian Federation

## Abstract

**Introduction:**

In recent years, there has been renewed interest in lithium therapy due to emerging evidence of the protective effects of lithium against neuronal death caused by a wide range of neurotoxic effects. Oxidative stress is a common pathway that is involved in various pathologies. In this regard, the development and study of new lithium compounds with combined antioxidant effects becomes relevant. Pyruvate has many potential benefits due to its positive effects on cellular metabolism.

**Objectives:**

The purpose of this study was to study lithium pyruvate on blood cells of healthy donors under conditions of induced oxidative stress.

**Methods:**

The study used blood from 20 healthy control group volunteers, aged 25 to 54 years. Venous blood was taken at baseline and then used for PBMCs extraction. After that cells were incubated during 24 hours in RPMI 1640 medium at 37°С and 5% carbon dioxide concentration. For oxidative stress induction hydroperoxide of trisubstituted butyl (HTB) was used in concentration of 50 μM. Cells were also incubated with lithium pyruvate in final concentration of lithium ions of 1.2 mM with or without HTB. Level of oxidative stress in culture was assessed by flow cytometer «Muse Cell Analyzer» (Merck Millipore, Germany) using «Oxidative stress» reagents kit (Merck Millipore, Germany). Statistical analysis was performed using the SPSS software, release 20.0 for Windows.

**Results:**

Percentage of cells with reactive oxygen species (ROS) cultivated with HTB (65,33 (41,95-79,30) %) was statistically significant higher compared to intact cells (11,03 (7,93-15,53) %) (p=0.001). After addition of lithium pyruvate in culture statistically significant antioxidant effects were observed. In PBMCs incubated with HTB and lithium pyruvate statistically significant decreased percentage of cells with ROS (42,70 (16,73-58,70) %) (p=0.001)

**Conclusions:**

A pronounced antioxidant effect of lithium pyruvate under induced oxidative stress on human peripheral blood mononuclear cells has been established. Lithium pyruvate can be considered as a promising psychotropic antioxidant for further experiments.

**Disclosure of Interest:**

None Declared

